# Neuroimaging and machine learning for studying the pathways from mild cognitive impairment to alzheimer’s disease: a systematic review

**DOI:** 10.1186/s12883-023-03323-2

**Published:** 2023-08-22

**Authors:** Maryam Ahmadzadeh, Gregory J. Christie, Theodore D. Cosco, Ali Arab, Mehrdad Mansouri, Kevin R. Wagner, Steve DiPaola, Sylvain Moreno

**Affiliations:** 1 School of Interactive Arts and Technology, Simon Fraser University, 250 – 13450 102 Ave, Surrey, BC Canada; 2 Gerontology Research Center, Simon Fraser University, Vancouver, BC Canada; 3 Oxford Institute of Population Ageing, University of Oxford, Oxford, UK; 4 Department of Computing Science, Simon Fraser University, Burnaby, BC Canada

**Keywords:** Systematic review, Alzheimer, Mild cognitive impairment, Prediction, Neuroimaging, Machine learning

## Abstract

**Background:**

This systematic review synthesizes the most recent neuroimaging procedures and machine learning approaches for the prediction of conversion from mild cognitive impairment to Alzheimer’s disease dementia.

**Methods:**

We systematically searched PubMed, SCOPUS, and Web of Science databases following Preferred Reporting Items for Systematic Reviews and Meta-Analyses (PRISMA) systematic review guidelines.

**Results:**

Our search returned 2572 articles, 56 of which met the criteria for inclusion in the final selection. The multimodality framework and deep learning techniques showed potential for predicting the conversion of MCI to AD dementia.

**Conclusion:**

Findings of this systematic review identified that the possibility of using neuroimaging data processed by advanced learning algorithms is promising for the prediction of AD progression. We also provided a detailed description of the challenges that researchers are faced along with future research directions. The protocol has been registered in the International Prospective Register of Systematic Reviews– CRD42019133402 and published in the Systematic Reviews journal.

**Supplementary Information:**

The online version contains supplementary material available at 10.1186/s12883-023-03323-2.

## Background

Alzheimer’s disease dementia (AD) is a slowly evolving neurodegenerative disorder that can begin 20 years or more before clinical diagnosis [1]. Through the progression of Alzheimer’s disease, multiple brain neurons and their connections are damaged or destroyed which affects an individual’s basic daily life functions such as walking, talking or swallowing [2]. The number of deaths due to AD has increased by 145% from 2000 to 2017 and is projected to increase further as the number of older adults (aged 65+) increases throughout the world [2]. While the risk of death due to Alzheimer’s disease raises with aging, currently, a disease-modifying/blocking treatment for severe AD is not available [3].

The early stages of AD comprise a symptomatic prodromal phase of dementia referred to as mild cognitive impairment (MCI) [4]. Ideally, treatments for AD would be initiated in this MCI stage before the subsequent accumulation of irreversible neurological damage [3]. However, not all individuals in this MCI stage will progress to AD, and a large number of research studies have attempted to develop models capable of identifying those at high risk of progressing from MCI to AD dementia from those at low risk [4–6]. If successful, this would help researchers target individuals who would most benefit from prospective therapies with early interventions and clinical trials.

In the last decade, with advances in the analysis of neuroimaging data, many studies have shown the potential of these modalities in facilitating the detection of AD in its early stages as well as the prediction of clinical progression to AD [7–12]. In this regard, the present review includes data acquired using positron emission tomography (PET), structural magnetic resonance imaging (MRI), functional MRI, Single Photon Emission Computed Tomography (SPECT), and electroencephalography (EEG) modalities, which are common techniques for the acquisition of functional and anatomical brain data. Given that each of these modalities has certain advantages and limitations, some researchers have turned to use combinations of different modalities to obtain a more comprehensive set of neurological data; these studies are also included in the present review. However, due to the high complexity of the brain function and structure, there are still unresolved challenges regarding the efficient techniques to integrate multiple measures and modalities that are associated with the transition to AD dementia.

Several data analysis approaches including machine learning and deep learning algorithms have been developed to automatically analyze and quantify brain function, structure, morphology, and connectivity to identify individuals at risk of converting from MCI to AD [13–15]. Recent years have seen considerable growth in the number of machine learning (ML) algorithms (i.e., support vector machine, random forest, ensemble multiple kernel learning, K-nearest neighbor) that have been developed for this, using high-dimensional clinical and biomarker data [5, 16, 17]. In particular, ML algorithms can learn fine and complex patterns of change that occur across the neuroimaging modalities during the process of progression from MCI to AD dementia. However, the effective clinical translation of these approaches still requires a generalizable model that can be applied in real-life settings with the general public.

Consequently, the overarching objective of the present work is to provide a systematic review of recently published studies focused on the prediction of conversion from MCI to AD dementia using neuroimaging modalities and machine learning approaches. Our review will synthesize two main areas of active research: (i) an exploration of the most recent neuroimaging modalities used to predict MCI individuals who progress to AD, and (ii) a systematic discussion of machine learning techniques applied to identify the changes of factors contributing to the transition from MCI to AD dementia.

## Methods

### Study protocol

The methods of this systematic review have been conducted in accordance with the PRISMA guidelines for systematic reviews [18]. The protocol has been registered in the International Prospective Register of Systematic Reviews– CRD42019133402 and published in the Systematic Reviews journal [19].

“Systematic review protocol is like a roadmap that provides in advance information on the objectives, hypothesis, and methods of reviewing the published studies. This assists in outlining the whole process of systematic review in a transparent way. However, in the review discussion, the results of the study are evaluated, interpreted, and explained. The relationships between the findings and literature review, and arguments in support of the conclusions are discussed. Furthermore, limitations of the study and recommendations regarding the future pathway are provided.”

### Search strategy

A systematic search was performed across three electronic databases: PubMed, Web of Science, and Scopus. Because the present review paper focuses on a cutting-edge research topic that is developing quickly, we restricted our review to a recent window of time from January 1st, 2017 to March 1st, 2019. The search strategy for PubMed/MEDLINE database is presented in Additional file 1. The search string was adapted to the search mechanisms of each of the selected databases. We further hand-searched the reference lists of included articles to identify additional studies to complement the electronic searches.

### Study selection

Two authors (MA and KRW) independently screened the titles and abstracts of all articles identified from the initial search according to the inclusion/exclusion criteria. If the relevance of an article was unclear from its title and abstract, both reviewers examined the full text of the paper in detail. Conflicts between the two authors were resolved through discussion, and in case of disagreement, a third reviewer (TDC) was consulted to make the final decision. For the present review, the following inclusion criteria were used: (1) all studies had to address research on Alzheimer’s disease and MCI; (2) all studies had to have focused on the prediction of conversion from MCI to AD; (3) all studies had to describe the results in a way that allowed us to extract the accuracy, sensitivity and/or specificity of the method at predicting conversion to AD, for comparison purposes; (4) we included those studies that described their methods in sufficient detail to enable replication; (5) we included all studies that used at least one type of neuroimaging modality in their research; and (6) all studies had to be published between January 1st, 2017 and March 1st, 2019. The current review paper has focused on a cutting-edge research topic that is fast paced; therefore, we limited the search to examine only recent studies. Studies with any of the following characteristics were excluded: (1) articles addressing research on other types of dementia such as Frontotemporal dementia, Lewy body dementia, Vascular dementia, Huntington’s disease, and Parkinson’s disease and mixed dementia; and (2) ineligible or non-peer-reviewed studies such as conference proceedings, editorial, secondary data analyses, review articles, and book reviews. We did not impose language restrictions on the search and translated articles when necessary.

### Data extraction

Using a predefined standardized template to extract data from the included studies, we investigated research on the prediction of progression from MCI to AD dementia using biomedical image processing with machine learning techniques. Thus, we explored various modalities that have been used in the selected studies as well as data analysis techniques in the early prediction of AD dementia. In this context, the two aforementioned reviewers extracted the following data: (1) author(s); (2) year of publication; (3) source of data; (4) follow-up period (conversion period); (5) sample size (number of participants with MCI, stable MCI, and progressive MCI); (7) modalities; (8) neuroimaging feature; (9) data analysis techniques and (10) performance of results including accuracy, sensitivity, specificity. Additionally, If the information in a study were not unclear or missing, we contacted the authors for clarification.

### Data synthesis

In instances where meta-analysis was not possible, we aimed to explore heterogeneity descriptively using structured narratives and summary tables. Therefore, a narrative synthesis method was used to describe the results of the identified studies. In this context, we provided a descriptive summary focused on the investigation of neuroimaging modalities, features, data resources and data analysis techniques, and performances in terms of accuracy, sensitivity, and specificity. Data synthesis could help us to identify gaps in the evidence, areas of strength and fields in need of improvement related to methodological development and biomarkers identification to achieve the main goal of prediction of progression from MCI to AD.

## Results

### Study selection

**Fig. 1:**
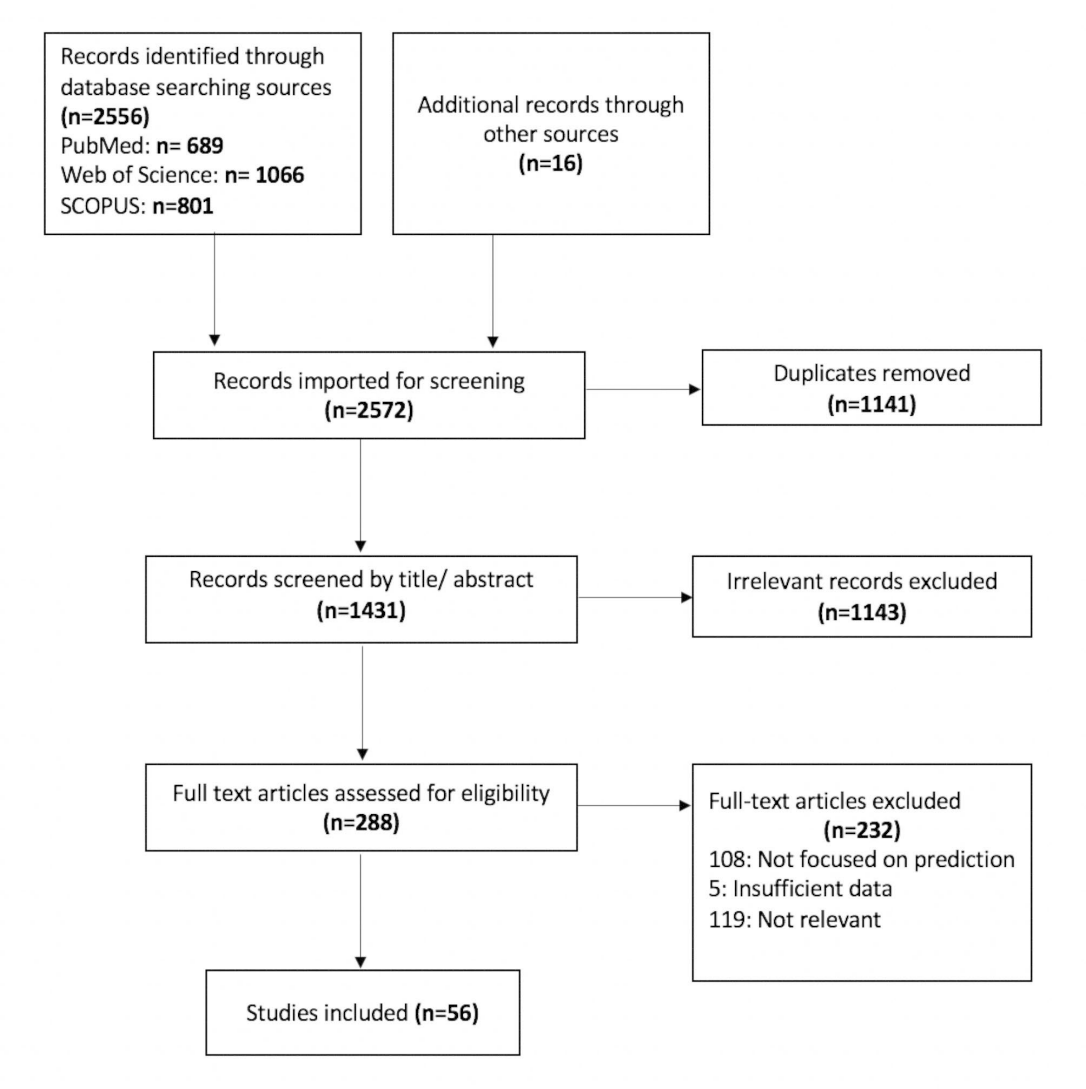
Flowchart of the systematic review process

The initial search of three databases identified 2556 articles. An additional 16 articles were identified by hand-searching the articles’ references and other sources. After eliminating duplicate results, this was reduced to 1431 articles for prospective inclusion. Next, the titles and abstracts of these articles were screened based on the exclusion/inclusion criteria. At this step, we excluded 1141 articles, leaving a total of 288 papers for a full-text screen. After reading the full texts of these 288 articles, 56 articles were found that met the selection criteria. Figure 1 provides a flowchart of the identification and selection of eligible studies.

### Neuroimaging modalities

In this section, a detailed explanation of the most recent neuroimaging modalities used to predict the progression from MCI to AD dementia is provided. Furthermore, Table 1 reports an overview of the modalities used in the included articles as well as the number of studies that used each single modality or the combination of neuroimaging modalities.

#### MRI modalities

Out of all 56 identified articles, structural MRI was the most commonly used neuroimaging modality to predict the conversion from MCI to AD (48.2%). While only one study developed a multi-label prediction approach with both baseline sMRI and cerebrospinal fluid (CSF) data [20], five studies (8.9%) investigated the progression to AD among MCI patients by combining the structural neuroimaging with baseline neuropsychological assessments (NPA) (i.e. Mini-Mental State Exam (MMSE) [21]), Alzheimer’s disease assessment score (ADAS), or logical memory immediate recall (LIMM) [16]). One study evaluated the combination of three modalities (sMRI, CSF, and NPA) to identify patients with MCI who are most likely to progress to AD dementia within a relatively short period. It showed that there is a gradual improvement in the statistical measures across increasing biomarker combinations [22]. A single study added demographic information and genetic factors to the previous three modalities [23]. In this study, an integrative framework was developed that combined cross-sectional neuroimaging biomarkers measured by MRI such as hippocampal volume and entorhinal cortical thickness, demographic information including age, sex, years of education and Apolipoprotein E (APOE) genotype at baseline with longitudinal CSF and cognitive performance biomarkers including composite scores for executive functioning (ADNI-EF) and memory (ADNI-MEM) [23]. A composite model including sMRI features, APOE genotype, neuropsychological assessments, and demographic information was assessed in two separate studies [24, 25]. Lastly, one study demonstrated the potential of using resting-state functional MRI (rs-fMRI) to classify MC converters-to-AD from non-converters with high accuracy by identifying affected brain regions underlying the conversion [11]. The majority of studies have been done with structural MRI scans. While these systems are known for providing high spatial resolution for neuroimaging data, they are immobile and require expensive ongoing maintenance. Several studies that combined MRI scans with neuropsychological assessments or demographic information and genetic factors showed the potential improvement of model performance.

#### PET modalities

Out of 56 studies, six research studies used PET in predicting the conversion to AD dementia in patients with MCI. Four out of these six studies assessed the capacity of fluorodeoxyglucose PET (FDG-PET) in discriminating MCI patients who converted to AD from those who did not by capturing the early deterioration of brain glucose metabolism. One of these six studies showed that their proposed deep learning approach could accurately predict cognitive decline in MCI patients by combining information from FDG-PET and 18 F-florbetapir (AV-45) PET [26]. Another study explored both separate and combined values of brain glucose metabolism (FDG-PET) and cerebral amyloid burden (Pittsburgh Compound-B-PET) for the selection of subjects who would eventually convert to AD dementia [27]. Their findings showed that measures of brain glucose metabolism and amyloid load provide powerful information with complementary roles that could improve the global accuracy of AD conversion prediction [27]. The efficiency of combined neuropsychological assessments (ADAS-cog score), amyloid PET (florbetapir PET), and FDG PET modality over single modalities in the prediction of AD conversion was evaluated in only one study [28]. Finally, one study used amyloid PET imaging along with demographic information such as age, gender, and APOE genotype status to identify incipient AD dementia individuals using a machine learning-based probabilistic method [29].

#### EEG and SPECT modalities

EEG is a non-invasive and relatively low-cost procedure that has been widely used in a large number of studies focused on pathological brain aging [30]. However, our selection criteria as explained in Sect. 2.2 identified only a single study that, using EEG connectivity markers, investigated the conversion of prodromal MCI to AD [10]. Their experimental results indicated that EEG connectivity analysis, combined with APOE genotyping, represents a combination of biomarkers that reach high classification performance for those at high-risk of converting from MCI to [10].

Likewise, a single study was found that evaluated the ability to predict AD dementia by considering the baseline of Single Photon Emission Computed Tomography (SPECT) perfusion abnormalities along with long-term memory disorders [31]. Compared to FDG- PET, SPECT is relatively more accessible and cost-effective [32, 33]. The results confirmed that the association of neuropsychological (EMS) and brain perfusion SPECT could develop the accuracy in detecting subjects who will progress to AD dementia [31].

#### Neuroimaging multimodalities

The inclusion of additional biomarkers may improve the accuracy with which individuals at risk of converting from MCI to AD are identified. We identified seven studies (12.5%) that investigated the predictive power of combining multimodal information from sMRI and PET images. Two of these studies only considered the combination of these neuroimaging data [15, 34], and the remaining five studies also added other non-neuroimaging biomarkers, including neuropsychological assessment, demographic information (i.e. gender, age), biomarkers from cerebrospinal fluid (CSF), genetic factors and cognitive resilience (i.e. the number of errors on the American National Adult Reading Test (ANART)). Some of these studies showed that the performance of classifying progressive-MCI from stable-MCI individuals using a combination of modalities was better than those built with a single modality [34, 35]. A study explored the integration of two types of MRI data; rs-fMRI and sMRI. Their findings also revealed that compared to a single modality approach, the integration of rs-fMRI and sMRI can improve the performance of identification of the early stage of AD [36].

**Table 1:** Summary of modalities used in the prediction of conversion from MCI to AD dementia as well as the number of studies used each modality or the combination of modalities

Neuroimaging Modality	Non- Neuroimaging Modality	Number of Studies	Ref
sMRI	NA	28	[13], [37], [38], [39], [40], [41], [42], [6], [43], [44], [45], [46], [47], [48], [17], [9], [49], [50], [51], [52], [53], [54], [55], [56], [57], [58], [59].
	CSF	1	[20]
	NPA	5	[21], [16], [60], [5], [61]
	CSF, NPA	1	[22]
	NPA, genetic, Demo	2	[24], [25]
	CSF, NPA, genetic, Demo	1	[23]
fMRI	Rs-fMRI	1	[11]
SPECT	NPA	1	[31]
EEG	Gen	1	[10]
PET	NA	6	[26], [27],[62], [8], [63], [64]
	NPA	1	[28]
	genetic, Demo	1	[29]
sMRI, PET	NA	2	[34], [15]
	NPA, Demo	1	[65]
	NPA, genetic, Demo	1	[66]
	CSF	1	[67]
	CSF, NPA	1	[35]
	CSF, NPA, genetic, Demo, Cognitive resilience	1	[7]
sMRI, fMRI	NA	1	[36]

**Abbreviation:** CSF, Cerebrospinal Fluid; NPA, Neuropsychological Assessment; SPECT, Single Photon Emission Computed Tomography; sMRI, structural Magnetic Resonance Imaging; rs-fMRI, resting-state functional MRI; EEG, Electroencephalogram; PET, Positron Emission Tomography; Demo, Demographic information; NA, Not applicable

**NOTE.** NA: Non- Neuroimaging Modality was not used in these studies

### Data analysis techniques

#### Conventional machine learning techniques

This section provides a systematic discussion of machine learning (ML) techniques applied to analyze the single/multimodal patient information in the process of transition from MCI to AD dementia. Table 2 illustrates different types of machine learning approaches with their validation system and performance including accuracy (ACC), sensitivity (SEN), and specificity (SPE) of predicting the incidence of AD along with neuroimaging modalities and extracted features.

As reported in Table 2, most ML methods used in the studies have a linear classification baseline. Regression is the simplest classification model, which assumes a linear relationship between the outcome variable and features. The evident disadvantages of regression are that it can miss nonlinear relationships between variables and that it is very sensitive to outliers. However, the major advantages of regression are the simplicity of its assumptions, its applicability to small datasets, and relatively high interpretability of results [68].

Table 2 also shows that from a total of 56 selected studies, Support Vector Machines (SVM) was the most frequently used ML technique (43.8%). SVM is a notable expansion of regression in the sense of detecting outliers and making robust linear models. However, the major drawback of SVM is that it can miss nonlinear relationships between features and outcomes [14]. Some examples of other techniques that appeared less frequently in the reviewed studies include Random Forest (RF), Ensemble Multiple Kernel Learning and Bayesian Rule.

While the majority of studies have used cross-validation as the model evaluation technique, eight of the studies did not report their validation techniques and one study did not use any validation system. The lack of using any validation system does not ensure the generalizability of their model to independent data that makes the reported evaluation unreliable.

The majority of the studies (80.3%, 45/56) used the Alzheimer’s Disease Neuroimaging Initiative (ADNI) database (adni.loni.usc.edu) in their models. This calls into question the applicability of these models to the general population, as these models may be overfitted to this dataset. The remaining studies used databases that were implemented in different countries including China [54], Germany [22], Italy [10, 31], Sweden [27], Korea [62], Belgium [35], and Finland [8].

**Table 2:** Summary of machine learning techniques in prediction of conversion from MCI to AD dementia

Ref	Year	Modality	Data base	Neuroimaging Features	Method(s)	Conversion Time (months)	Sample Size	Validation System	Performance (%)	Remark
[13]	2017	sMRI	ADNI	Textural features • Statistical features • Model based features • Image transforms Volumetric features • GM volume • WM volume • CSF volume	• Bayesian Rule • Majority Voting • RBF SVM	0–12	pMCI:30 sMCI:30 NC:30 AD:30	Leave-one-out	ACC:100 SEN:100 SPE:100	Bayesian Rule performed better than other classifiers. Feature selection: Gain ratio
[45]	2017	sMRI	ADNI	sMRI • GM volume • Cortical thickness • Surface area • Sulcal depth and curvature	SVM	0–24	pMCI:40 sMCI:46 NC:72	Leave-one out	ACC:98.84 SEN:97.5 SPE:100	Feature selection: L2-1 norm Regression
[22]	2017	• sMRI • CSF • NPA	Their database, Germany	Hippocampal volume	SVM	0–36	pMCI:28 sMCI:87	No report	AUC:82 SEN:89 SPE:67	-
[36]	2018	• sMRI • rs-fMRI	ADNI	sMRI • Volume of GM • Surface area • Cortical thickness • Curvature rs-fMRI • Local and global graph measures of brain connectivity	SVM	0–36	pMCI:18 sMCI:62	9-fold	ACC:96.97 SEN:94.97 SPE:100	Integrated sMRI with rs-fMRI.
[35]	2019	• sMRI • Florbetapir-PET • FDG-PET • CSF • NPA	Memory Clinic, Belgium	sMRI • Hippocampal volumes • WM hyperintensities • Cortical GM Florbetapir-PET • Cortical SUVR FDG-PET • PCC SUVR	Linear Mixed-Effect Model	0–12	MCI:49 NC:13 AD:16	independent test set	ACC:94 SEN:92 SPE:96	-
[56]	2017	sMRI	ADNI	sMRI • Stationary velocity field • Jacobian determinant • Divergence • Deformation • Geodesic length	SVM	0–36	sMCI:43 pMCI:67	10-fold	ACC:92 SEN:95 SPE:90	Dimensionality reduction: KPCA Feature selection: PCA
[10]	2018	•EEG •Genetic	Memory Clinic, Italy	EEG network small world properties of 7 frequency bands	Regression	38.17 ± 3.48* 18.29 ± 1.60*	pMCI:71 sMCI:74	10-fold	ACC:91.7 SEN:96.7 SPE:86	Feature selection: Kolmogorov-Smirnov test
[11]	2017	rs-fMRI	ADNI	Local and global measures of brain connectivity	SVM	6–36	pMCI:18 sMCI:62	9-fold	ACC:91.40 SEN:83.24 SPE:90.10	Feature selection: MRMR
[31]	2018	•Brain perfusion SPECT •NPA	Their database, Italy	Mean activity of ROIs	Ensemble Multiple Kernel Learning	0–24	pMCI:15 sMCI:27	No report	ACC:90 SEN:80 SPE:96	Feature selection: F-Score
[16]	2017	• sMRI • NPA	ADNI	Five volumetric features	Majority Voting Regression	0–36	sMCI:13 pMCI:16	Leave-one-out	ACC: 89.66 SEN: 87.50 SPE: 92.31	Volume features included entorhinal, fusiform, hippocampus, middle temporal lobe and ventricles.
[27]	2017	• FDG-PET • C-PiB-PET	Karolinska Institute, Sweden	FDG-PET • Manual labeling by visual inspection of AD positivity C-PiB-PET • SUVR score cut-off indicating amyloid positivity	Regression	0-26.5 (median)	sMCI:16 pMCI:14	No report	AUC: 96	-
[51]	2017	sMRI	ADNI	Morphological features: • Whole-brain GM, WM • Subcortical structures • Cortical lobes	SVM	0–36	sMCI:132 pMCI:95 NC:135 AD:65	10-fold	ACC:88.99 SEN:86.32 SPE:90.91	Considering the amygdala or hippocampus as ROI performed better than other features.
[8]	2017	FDG-PET	Their dataset, Finland	13 meta-VOI-based FDG uptake in each hemisphere	SVM	7.5 ± 1.5 (years)	pMCI:95 sMCI:27 NC:42	21-fold	ACC:88.5 SEN:87.4 SPE: 92.6	SVM model used age-corrected baseline data.
[65]	2017	•FDG-PET •sMRI • NPA •Demo	SEAD-J	sMRI • z-score in medial temporal structures including entorhinal cortex, hippocampus, and amygdala FDG-PET • Visual inspection of PET images (predefined patterns)	Regression	0–60	sMCI:19 pMCI:49	No report	ACC: 87.9 SEN: 89.8 SPE: 82.4	Feature Selection: Stepwise
[24]	2018	• sMRI • NPA • Genetic • Demo	ADNI	• Hippocampal occupancy • Eight texture features	3D Texture Analysis	0–36	pMCI:98 sMCI:106 NC:225 AD:183	No report	SEN: 86.7 SPE: 83	3-D voxel-based texture analysis of MR images calculated texture features.
[66]	2017	• sMRI • FDG-PET • Genetic •Demo • NPA	ADNI	sMRI • GM volume • SUVR of several brain networks FDG-PET • SUVR of brain regions	Regression	0–36	pMCI:126 sMCI:108 AD: 121 NC:120	Independent test set	ACC: 84.62 SEN: 86.51 SPE:82.41	Feature Selection: ICA, Cox Proportional Hazard
[60]	2018	• sMRI • NPA	ADNI	• Volumes of brain regions • Surface area • Cortical thickness	Autoregressive SVM	0–36	pMCI 54 sMCI:65	5-fold	ACC: 84.29 SEN: 70.36 SPE: 92.31	-
[29]	2017	•Florbetapir PET • Demo • Genetic	ADNI	Regional PET SUVR intensities	Random Forest	0–24	pMCI:43 sMCI:230	Independent test set	ACC:84 SEN: 70.8 SPE: 86.5	Feature Selection: Voxel-wise Logistic Regression
[61]	2017	• sMRI • NPA	ADNI	Voxel-wise MRI features	Random Forest	0–36	pMCI:171 sMCI:129 NC:229 AD: 191	10-fold	AUC:83.8 SEN:85.2 SPE:71.8	Feature Selection: Elastic Net, LASSO
[5]	2017	• sMRI • NPA	ADNI	Fuzzy sets of hippocampal volume	SVM	0–36	pMCI:86 sMCI:71 NC:115 AD:96	10-fold	ACC:83.4 SEN:87.2 SPE:78.9	Feature Selection: Kruskal-Wallis test
[7]	2019	•sMRI •FDG-PET •Florbetapir-PET •CSF •NPA •Cognitive resilience •Genetic •Demo	ADNI	sMRI • Hippocampal subfields volume • Cortical thickness FDG-PET • Degree of glucose metabolism in AD-specific ROIs F-florbetapir PET • Regional amyloid deposition assessed by SUVR	SVM, MKL, GLM	0–36	pMCI:39 sMCI:96	5-fold	ACC:81 SEN:93 SPE:77	SVM performed better than other classifiers. Feature Selection: Mutual Information
[54]	2019	sMRI	XuanWu Hospital, China	Whole-brain WM structural networks/connectome	Kernelized SVM	0–24	pMCI:26 sMCI:26 NC:26	Leave-one-out	ACC: 80.77 SEN:92.31 SPE:69.23	Feature Selection: ANOVA, T-Test
[25]	2017	• sMRI • NPA • Demo • Genetic	ADNI	• Hippocampal volumetric integrity • Corpus callosum • Circularity	Random Forest	0–6	pMCI:162 sMCI:85	10-fold	ACC:80.2 SEN:79.6 SPE:81.2	-
[44]	2018	sMRI	ADNI	• Voxel-based morphometry • Hippocampus volumes • Volumes of entorhinal cortex • Set of regional volumetric • Surface area • Cortical thickness	SVM, RLR	0–36	sMCI:100 pMCI:164 AD:200 NC:231	Nested 10-fold	ACC:79.58 SEN:84.07 SPE:51.5	Compared to RLR, SVM yielded lower specificity. Feature Selection: Elastic Net, PCA
[47]	2017	sMRI	ADNI	MRI Voxels at three levels (voxel, patch, and image level)	Hierarchical classification based on LRC	0–48	sMCI: 61 pMCI: 70	Two nested CV loops (10-fold for each loop)	ACC: 79.4 SEN:86.5 SPE: 78.2	Feature Selection: Regression
[62]	2018	FDG-PET	Seoul National Hospital, Korea	Degree of cerebral glucose metabolism in 6 ROIs	Regression	0–24	pMCI:19 sMCI:35	No validation	AUC:79.4 SEN:76.47 SEP:75	Both visual rating and computer-assisted analysis showed similar accuracy.
[17]	2018	sMRI	ADNI	• Structural volumes • Atrophy rates	SVM, Random Forest	0–24	pMCI:177 sMCI:166	6-fold	ACC: 79 SEN:82 SPE:74	SVM and Random Forest performed similarly.
[21]	2018	• sMRI • PHS • NPA	ADNI	Volumetric features of ROIs	Linear Mixed-Effect Model	0–36	sMCI:154 pMCI:182	No report	ACC: 78.9 SEN: 79.9 SPE: 77.8	Feature Selection: Cox Proportional Hazards Model
[52]	2018	sMRI	ADNI	SBM texture measures of GM and WM	SVM	6–12	sMCI:64 pMCI:70 AD: 99 NC: 122	10-fold	ACC:77.6 SEN: 72.9 SPE: 82.6	Feature Selection: Lasso, T-Test
[58]	2018	sMRI	ADNI + Their own database, China	Morphological features • Cortical thickness • Surface areas • GM volume • Sulcal depth • Metric distortion • Mean curvature	RBF SVM	0–24	pMCI:84 sMCI:86 NC:169	Leave-one-out	ACC: 77.06 SEN: 77.91 SPE: 76.19	Feature Selection: LASSO
[28]	2018	•FDG-PET •Florbetapir-PET •NPA	ADNI	Amyloid and metabolic SUVRs at voxel level and cortical ROIs	SVM	37 ± 14	pMCI:85 sMCI:204 NC:251 AD:144	Leave-one-out	ACC:77 SEN:74 SPE:78	Correlation between amyloid and metabolic SUVRs was assessed using Pearson’s coefficient.
[20]	2018	• sMRI • CSF	ADNI	GM volume	SVM	0–24	pMCI:86 sMCI:106 NC: 112 AD: 109	10-fold	ACC: 76.3 SEN: 73.4 SPE: 78.6	Feature Selection: Transfer Learning
[46]	2018	sMRI	ADNI	Hippocampus texture	SVM	No report	pMCI:165 sMCI:223 NC:226 AD:186	Stratified 10-fold	AUC:76.1 SEN:74.9 SPE:70.2	Feature Selection: Histogram
[38]	2017	sMRI	ADNI	Raw voxel value of regions with GM atrophy	SVM	0–36	pMCI:71 sMCI:65 NC:94 AD:92	10-fold	ACC:75 SEN:76.92 SPE:73.23	Feature Selection: T-Test, Genetic Algorithm
[67]	2017	• sMRI • PET • CSF	ADNI	sMRI • Volume of GM tissue in 93 ROIs PET • Mean intensity of ROIs	SVM	0–18	pMCI:167 sMCI:226 NC:186 AD:226	10-fold	ACC:74.58 SEN:51.31 SPE:88.71	Feature Selection: L2-1 norm Regularization
[53]	2018	sMRI	ADNI	Local surface roughness of hippocampus	Regression	0–36	pMCI:36 sMCI:61	Nested	ACC:74.3 SEN:77.4 SPE:72.3	Feature Selection: ANOVA
[50]	2018	sMRI	ADNI	ROI features & interregional features	Ensemble Multiple Kernel Learning	0–18	pMCI:120 sMCI:160 NC:230 AD:200	10-fold	ACC: 74.28 SEN:71.51 SPE: 76.46	Feature Selection: F-Score
[15]	2017	• sMRI • PET	ADNI	sMRI • Average intensity of each ROI PET • GM tissue volumes in ROIs	SVM	0–18	pMCI:43 sMCI:56 NC:52 AD:51	10-fold	ACC: 72.4 SEN:49.1 SPE:94.6	Feature Selection: L2-1 norm Regularization
[63]	2018	FDG-PET	ADNI	• Statistical features • Connectivity features • Graph-based features	Ensemble SVM	0–24	pMCI:44 sMCI:44 AD:94 NC:90	Nested 10-fold	ACC: 72.33 SEN: 73.27 SPE:73.11	Feature Selection: LASSO
[49]	2018	sMRI	ADNI	Texture features of the ROIs and spatial correlations	Multiple Kernel Learning	0–18	pMCI:120 sMCI:160 NC:230 AD:200	10-fold	ACC: 72.08 SEN: 75.11 SPE: 71.05	Feature Selection: F-Score Feature Extraction: Whole brain hierarchical network
[40]	2017	sMRI	ADNI	• Cortical thickness • Cortical surface area • Cortical volume • Sub-cortical volume • both hemisphere SA volume • Total intracranial volume	K-Nearest Neighbor	No report	pMCI:168 sMCI:229 NC:229 AD:192	10-fold stratified	ACC: 70.4 SEN: 67.7 SPE: 71.8	Feature Selection: MFA, MKL
[43]	2017	sMRI	ADNI	• Regional cortical thickness • Subcortical volumes	Partial Least Squares Regression	0–36	pMCI:70 sMCI:75 NC:228 AD: 195	No report	ACC: 68.3 SEN:80 SPE:57.3	Age correction was added to improve the performance.
[48]	2018	sMRI	ADNI	CVRS measures including the scales of hippocampal atrophy, cortical atrophy, ventricular enlargement, and small vessel disease	Random Effect Model	0–36	sMCI:271 pMCI:69	No report	AUC:67.7 SEN:63.8 SPE:65.7	Feature Selection: Cox Proportional Hazards Mode
[59]	2018	sMRI	ADNI	Morphological features • Cortical thickness • Surface area • Volume • Local gyrification index • Sulcal depth • Gyrus height	SVM-RFE	0–36	MCI:221 NC:165 AD:142	N-fold (N = 5,7,10)	ACC: 67.42 SEN: 72.22 SPE: 61.05	Feature Selection: Recursive Feature Elimination, LASSO
[55]	2018	sMRI	ADNI	GM density	SVM	0–18	pMCI:76 sMCI:134 NC:162 AD:137	5-fold	ACC:65.4 SEN: 68.3 SPE: 64.2	Feature Selection: LASSO
[57]	2019	sMRI	ADNI	Cortical thickness of brain regions	SVM	0–36	pMCI:126 sMCI:95 NC:165 AD:142	10-fold	ACC: 63.69 SEN: 78.56 SPE: 45.39	Feature Selection: Manifold Regularization, L2-1 norm Regularization
[41]	2017	sMRI	ADNI	Voxel-wise tissue probability maps of WM, GM, and CSF	MARS Regression	0–18	pMCI:176 sMCI:134 NC:162 AD:136	3-times repeated k-fold	SEN: 62.16 SPE:59.70	Feature Selection: Generalized Linear Model, SVM, L2-1 Norm
[39]	2017	sMRI	J-ADNI	Connectivity matrix of GM	SVM	6–36	pMCI:45 sMCI:42 NC:61 AD:83	10-fold	ACC:61.05 SEN:52.65 SPE:70.52	Feature Selection: Histogram

**Abbreviations:** CSF, Cerebrospinal Fluid; NPA, Neuropsychological Assessment; SPECT, Single Photon Emission Computed Tomography; sMRI, structural Magnetic Resonance Imaging; rs-fMRI, resting-state functional MRI; EEG, Electroencephalogram; PET, Positron Emission Tomography; C-PIB-PET, C-labelled Pittsburgh Compound-B- Positron Emission Tomography; Demo, Demographic information; RBF, Radial Basis Function; J-ADNI, Japanese-Alzheimer’s Disease Neuroimaging Initiative; MFA, Marginal Fisher Analysis; MKL, Multiple Kernel learning; GLM, Generalized Linear Models; MARS, Multivariate Adaptive Regression Splines; RLR, Regularized Logistic Regression; SUVR, Standardized Uptake Value Ratio; Random under sampling Random Forest; ROI, Regions of Interest; ADNI, Alzheimer’s Disease Neuroimaging Initiative; pMCI, progressive Mild Cognitive Impairment; sMCI, stable Mild Cognitive Impairment; GM, Grey Matter; WM, White Matter; SEAD-J, Studies on Diagnosis of Early Alzheimer’s Disease-Japan; SBM, Spherical Brain Mapping; MRMR, Multivariate Minimal Redundancy Maximal Relevance; KPCA, Kernel Principle component Analysis; LASSO, Least Absolute Shrinkage and Selection Operator; CV, Cross Validation; PCC, Posterior Cingulate Cortex; STD, Standard Deviation; RFE, Recursive Feature Elimination

**NOTE.** Modality: the type of modality in each study. Neuroimaging Features: neuroimaging features in each study. Conversion Time: the time range for conversion from MCI to AD dementia. Performance: performance of classifying sMCI vs. pMCI in terms of accuracy (ACC), sensitivity (SEN), specificity (SPE), and area under the curve (AUC) when available

**NOTE.** Several studies focused on the discrimination of pMCI from sMCI as well as binary (i.e., AD vs. NC) or multiclass classification (i.e., AD vs. MCI vs. NC). In this study, we only focused on the classification of pMCI from sMCI and the performance section indicates the best results for this type of classification

*Months of follow-up for sMCI and pMCI groups were 38.17 ± 3.48 and 18.29 ± 1.60, respectively

#### Deep learning techniques

Deep learning (DL) methods are a family of machine learning algorithms that are widely used in medical imaging due to their specific architecture [64]. Compared to conventional machine learning techniques that often have a “shallow” structure, deep learning methods are composed of several layers that integrate and abstract information at each layer [6]. Besides, in deep learning algorithms, feature learning and the discovery of informative representations from data are performed automatically [69]. As such, DL methods can easily handle complex nonlinear data without the requirement of the professional knowledge of experts in the field. However, they are usually dependent on large training data and can have very low interpretability [6].

The list of deep learning techniques, along with validation techniques, modalities, and neuroimaging features, are indicated in Table 3. This table shows that deep learning techniques were used in eight studies and consisted of Feed-Forward Neural Networks (NN) [9, 34, 64], recurrent Neural Networks (RNN) [23], Convolutional Neural Networks (CNN) [26, 37, 42], and a combination of CNN and RNN [6].

Feed-Forward NN is a multi-layer architecture where each layer consists of a group of neurons. A neuron can be imagined as logistic regression. The NN is capable of learning complex relationships by utilizing non-linear activation functions. One research group proposed a Multiscale Deep Neural Network (MDNN) to exploit the multiscale features [34]. They applied their algorithm independently on single FDG-PET, single sMRI images and the combination of both modalities. Their findings showed that the performance of their multiscale neural network built with the combination of FDG-PET and structural MRI image modalities yielded a higher classification performance compared to using either structural MRI or FDG-PET scans alone [34]. However, the drawback of this architecture was that it required a high number of parameters, making the training task challenging.

Another study used a multimodal RNN to study the longitudinal cerebrospinal fluid (CSF) and cognitive measurements that are integrated with cross-sectional MRI biomarkers. An RNN has feedback loops on the hidden neurons, which can capture sequential information and share parameters across different time points. In this study, the architecture of their designed RNN allowed them to achieve a high prediction accuracy by incorporating longitudinal multi-domain data. However, their approach was limited by the small number of subjects as they needed to have all the required modalities at all time points [23].

A Convolutional neural network was used in three studies. In CNN, the building blocks are sets of filters that can be used to extract both local and high-level features from the input using convolution operation. Features are learned based on a hierarchical framework, starting with simple features such as edges and shapes, and going through more complex and detailed patterns at later stages of the network [70]. In the reviewed studies, this characteristic of CNN helped them in capturing the spatial relations between image pixels, resulting in higher performance. For example, in one study the designed CNN algorithm was robust to the variability of imaging protocols and qualities [37] and their results showed the feasibility of deep learning to capture the full spectrum of heterogeneity among data. This provided a less dataset-specific approach for developing a highly accurate predictive model.

In the last study, using the combination of convolutional and recurrent neural networks provided the opportunity to learn both spatial and longitudinal features of structural MR images at multiple time points [6]. Their results showed promising performance for longitudinal analysis of AD prognosis using the combination of RNN and CNN methods.

**Table 3:** Summary of deep learning techniques in prediction of conversion from MCI to AD dementia

Ref	Year	Modality	Data base	Neuroimaging Features	Conversion Time (months)	Sample size	Method/ Network Structure	Validation System	Performance (%)	Remark
[26]	2018	•FDG PET •AV-45 PET	ADNI	CNN feature map obtained from the PET images	0–36	pMCI:79 sMCI:92 NC:182 AD:139	CNN	10-fold	ACC:84.2 SEN:81 SPE:87	Deep CNN was trained using 3-D PET volumes of AD and NC as inputs to discriminate between sMCI and pMCI.
[34]	2018	• FDG-PET • sMRI	ADNI	sMRI •Patch-based volume FDG-PET •Patch-based mean intensity	0–36	pMCI:217 sMCI:409 NC:378 AD:238	Multiscale Multimodal DNN	10-fold	ACC: 82.93 SEN:79.69 SPE: 83.84	-
[64]	2018	FDG-PET	ADNI	Patch-based mean intensity	0–36	pMCI:112 sMCI:409 NC:304 AD:226	Multiscale DNN	10-fold	ACC: 82.51 SEN: 81.36 SPE: 82.85	Ensemble multiple classifiers using different validation set made the network more robust and stable.
[9]	2018	sMRI	ADNI	•Morphology of Hippocampus • Cortical volume • Surface area • Cortical thickness	0–36	pMCI:164 sMCI:100 NC: 229 AD:188	CNN	leave-one-out	ACC: 81.4 SEN: 89.6 SPE:68	-
[23]	2019	• sMRI •Demo • Genetic • NPA • CSF	ADNI	•Hippocampal volume • Entorhinal cortical thickness	0–24	pMCI:134 sMCI:561	RNN	5-fold	ACC: 81 SEN:84 SPE:80	-
[42]	2018	sMRI	ADNI	Local visual and global shape features of hippocampus	No report	pMCI:165 sMCI:231 NC: 223 AD:192	CNN	5-fold	ACC:75 SEN:73.33 SPE:76.19	Combined the global and local features of the hippocampus by 3D Densely CNN and shape analysis.
[37]	2019	sMRI	ADNI + Milan dataset, Italy	CNN feature map obtained from MRI	0–36	•ADNI pMCI:253 sMCI:510 NC:352 AD:294 •Milan pMCI:27 sMCI:23 NC:55 AD:124	CNN	10-fold	ACC:74.8 SEN:75.8 SPE:74.1	-.
[6]	2019	sMRI	ADNI	Spatial features from GM density map	0–36	pMCI:167 sMCI:236 NC:229 AD:198	CNN + RNN	5-fold	ACC:71.71 SEN:65.27 SPE:76.27	CNN learned the spatial features of MR images and then, RNN was constructed on the outputs of CNN.

**Abbreviations:** CSF, Cerebrospinal Fluid; NPA, Neuropsychological Assessment; Tomography; sMRI, structural Magnetic Resonance Imaging; PET, Positron Emission Tomography; Demo, Demographic information; ADNI, Alzheimer’s Disease Neuroimaging Initiative; pMCI, progressive Mild Cognitive Impairment; sMCI, stable Mild Cognitive Impairment; CNN, Convolutional Neural Networks; RNN, Recurrent Neural Networks; DNN, Deep Neural Network; VOIs, Volume of Interests; MLP, Multi-Layer Perceptron

**NOTE.** Modality: the type of modality in each study. Neuroimaging Features: Neuroimaging features in each study. Conversion Time: the time range for conversion from MCI to AD dementia. Performance: performance of classifying sMCI vs. pMCI in terms of accuracy (ACC), sensitivity (SEN), and specificity (SPE)

* The sMCI group remained as MCI throughout the ADNI study at the time of preparation of that manuscript (median follow-up time of 3 years) and the pMCI group progressed to AD with the median time to conversion of 1 year

## Discussion

The present systematic review sought to describe and evaluate the capability of existing neuroimaging procedures to predict the likelihood of individuals converting from MCI to AD dementia. In the MCI stage, brain damages are relatively slight and potentially reversible. Being able to accurately predict the progression to AD at the early stage of MCI could open important new therapeutic opportunities to improve the health outcomes of those most at risk of the disease [71].

To obtain a clearer understanding of the state of this work, we turned first to reviewing the neuroimaging methods and data sources currently being used to capture brain-based data as individuals progress through the pathway of MCI to AD. In terms of imaging modalities, the vast majority of recent work has been done with structural MRI and PET scans. While known for providing high spatial resolution for neuroimaging data, these systems are expensive, immobile and require ongoing maintenance. As a consequence, even if accurate prediction tools are developed using these modalities, they may see only limited use and applicability in screening those at risk for developing AD. By comparison, EEG is non-invasive, affordable and easy to implement the procedure. In real-world settings, where logistics and cost are important considerations, EEG may be a more desirable modality for screening MCI individuals. For example, low-cost EEG systems could allow more frequent testing to identify the progression of MCI over time. Unfortunately, our review identified only a single study that used this modality to study the progression of MCI to AD, and despite the highly accurate preliminary performance, there is clearly more work that needs to be conducted in this space [10, 72, 73]. The driving factor of this unbalanced reality is the fact that almost no longitudinal EEG datasets of MCI patients are available of MCI patients, whereas several longitudinal MRI datasets are currently available. We will turn to the consequences of this shortly but given the anticipated large increase in the rate of AD over the coming decade, there is likely insufficient time to conduct a new longitudinal study using EEG or another lower-cost neuroimaging modality. Nevertheless, we must stress that this remains a significantly under-developed field.

Our review also identified a noteworthy number of recent studies that combined MRI and/or PET scans with additional health data, such as neuropsychological assessments and genetic information [36, 37, 64]. Generally, these multi-modal studies led to better predictive accuracy, which would be expected given the complementary data captured by these different techniques. As before, however, the practical considerations of these multimodal approaches bear considering: a practical diagnostic solution becomes more difficult—and arguably less useful—as more data collection modalities are added. In the worst case, the necessary data might not be available and possible in practical settings [8]. Although the use of multiple modalities may be warranted at present in order to improve the accuracy of predictive models, we also encourage researchers to consider the practical implementation of these models in real-world use settings.

Continuing from this, our review identified a concerning trend with respect to the data being used to build these predictive models. The vast majority of the studies we reviewed (80.3%) used the Alzheimer’s Disease Neuroimaging Initiative (ADNI) database to study the progression from MCI to AD. Although this decision is understandable, given the volume and generally high quality of the data in this dataset, it has several limitations. First, in the ADNI database, most participants were recruited from memory clinics and advertisements, and MCI inclusion criteria were highly selective and the data was not sufficiently representative of the overall population [21]. Second, the ADNI is a multicenter study that has multiple acquisition protocols regarding the selection of patients, demographic characteristics, and diagnostic procedures. This results in systematic confounding effects and low statistical power, neither of which can be completely controlled for at the analytic level [8]. Lastly, the grouping criteria defined in the ADNI database are not perfect and contain false-categorized cases, and the study dropouts are biased toward high-risk individuals, all of which can lead to a bias in the estimates of progression rates. Thus, any models derived from this dataset will likely require subsequent validation studies before they can be used to predict the conversion of MCI to AD from the general public.

Furthermore, our review showed that most studies tracked the conversion from MCI to AD over a relatively short time frame ranging from about 6 to 36 months. Only a few studies had a longer follow-up duration. This relatively short follow-up period means that some of the individuals who were classified as stable MCI will in fact have progressed to AD dementia, or to some other neurodegenerative disease, beyond this tracking period. Therefore, the ground truth of clinically diagnosed sMCI patients might be inaccurate in ways that are difficult to quantify. Future studies should consider using databases with longer clinical follow-ups in order to exclude patients who do develop pMCI in the future.

As the data analysis algorithms used on these neuroimaging data are also critically important, this review also investigated the analysis approaches currently being used in the field as well.

Most studies used simple linear methods such as SVM and regression as their classification techniques. A justification for the prevalent use of linear methods over more sophisticated classification techniques is their simplicity, making them the first choice for such classification tasks. Additionally, the consistency of classifiers across different studies leaves the door open for direct comparison of other design decisions involved such as feature selection and preprocessing. However, these methods have well-studied limitations in their ability to accurately identify non-linear relationships between variables [74]. As non-linearities are relatively common in health data, we recommended that future studies turn to more advanced classification techniques that do not miss the nonlinear relationships between features and outcomes.

Deep learning algorithms are a family of ML methods that can learn hidden information from high-dimensional neuroimaging data. However, the main challenge of the deep learning methods used in the reviewed studies is that they rely on multiple layers of data processing with many parameters involved, and therefore require a large number of samples for training and tuning parameters. Particularly, this is a major issue in AD prediction, where the number of samples in the datasets available is limited [26].

ML methods can be categorized into those in which the feature selection is embedded with classifier construction, such as most DNNs [37], and those which usually use features transformed or features selected from original variables, such as Kernel-based SVMs [57]. Most of the reviewed methods required the features to be extracted from the image in a separate feature selection task before classification [75]. It is recommended that future studies consider using embedded models, such as CNNs, that allow the raw voxels to be fed into the network as the input. In fact, providing the neural network with raw data enables the network to learn the important features that could have been discarded during the feature selection. However, researchers should take into account that providing the network with raw data results in a higher computational cost due to the higher dimensionality of the inputs and also the extra tasks introduced for the feature extraction. Furthermore, in cases where valuable hand-engineered features can be defined based on domain knowledge, this approach may not be desirable because those valuable features and information provided by the experts will be missed.

In the reviewed studies, two major cross-validation techniques were used to evaluate accuracy: Leave-one-out cross-validation and 10-fold cross-validation. In the leave-one-out approach, one sample is retained from the method at each iteration and is used for testing; the rest of the samples are given to the method as training data. The process is repeated until all samples have been used for testing. In the 10-fold approach, samples are randomly partitioned into 10 groups of equal size; at each iteration, one group is used for testing and the remaining groups as training data. The process is likewise repeated until all groups have been used for testing. Both cross-validation methods are imperfect. On the one hand, 10-fold cross-validation carries additional computational cost over leave-one-out cross-validation and retains 10% of the available data from the method, which results in a potential underestimation of accuracy achievable by the method. On the other hand, leave-one-out cross-validation has a higher variance than 10-fold cross-validation with respect to the dataset. These two techniques are usually used because of their simplicity and the standardization of the test in comparing different methods. We encourage future works to use other classification methods with significant advantages over these two techniques, including nested cross-validations, bootstraps, holdout, and permutation tests. It should also be noted that some studies did not report their cross-validation approach, and presumably reported accuracy on their training set, which makes their reported evaluation unreliable.

In the present review, the performance of studies in terms of accuracy, sensitivity, and specificity are also represented. Although results are promising, these three measures can be limited in different ways by the data analysis technique being used. For example, several data analysis techniques are sensitive to feature selection and the tuning of many involved parameters. This problem is exacerbated where multiple layers of data processing algorithms are performed independently, and hence, the models and parameters cannot be jointly optimized. Furthermore, some of the discussed methods, such as deep learning models, are black-box models, meaning that it is difficult to understand why and how they make their decisions. This leads to limitations in the interpretability of the results. For example, it is difficult to infer which features and to what extent are contributing to the decision made by the classifier in deep learning models. In future research, these limitations should be addressed in order to develop more reliable predictive models that can be used in routine clinical settings.

It should be noted that most of the studies are conducted by researchers in the field of computer science and they have focused on developing their data analysis algorithms using commonly used types of modalities and features. Thus, in most of these studies, a systematic search to find the appropriate modalities and feature(s) is ignored. In the future, more collaboration among researchers in the field of computer science, neuroscience and cognitive science would assist to overcome the challenges and achieve a desirable performance by merging these domains.

There are a few limitations to the current systematic review. One limitation is that, due to differences in datasets, length of follow-up, type of modalities, prediction models, sample sizes and validation methods, it was not feasible to quantitatively compare the performances of the reviewed studies and determine the “best” approach for predicting the conversion likelihood from MCI to AD. Another limitation was the lack of detailed reports of features, validation methods, data analysis techniques, and data sources in some of the MCI conversion studies. However, we provided a comprehensive review of the strengths and drawbacks of neuroimaging modalities and data analysis techniques used in the reviewed studies along with concrete recommendations for future studies.

## Conclusion

A systematic review of neuroimaging procedures for the prediction of transition from MCI to AD dementia was conducted to describe their neuroimaging modalities and features as well as associated machine learning techniques in the most recent studies. With respect to data modality methods, the vast majority of current studies are using MRI data from the ADNI dataset. As a result, the field is at risk of developing models with reproducibility with the general public, and which may lead to methods that are too cost-prohibitive and logistically challenging to be of broad use. With respect to data analysis methods, deep learning approaches for analyzing brain scans could achieve high performance. There are major concerns regarding the use of nonrepresentative participants, clinical follow-ups, standardization of protocols and technical elements of diagnostic tests, and dealing with dropouts. The finding of studies would be more valuable if the larger sample size and longer conversion period could be considered. These issues need to be resolved before predictive models can be deployed in healthcare settings for the identification of individuals at risk of progressing from MCI to Alzheimer’s disease.

### Electronic supplementary material

Below is the link to the electronic supplementary material.

Supplementary Material 1

## Data Availability

Data sharing is not applicable to this article as no datasets were generated or analyzed during the current study.
