# Supplementary material for: Neuroimaging and machine learning for studying the pathways from mild cognitive impairment to alzheimer’s disease: a systematic review
Source: BMC Neurol. 2023 Aug 22;23:309. doi: 10.1186/s12883-023-03323-2 (PMC10463866; doi:10.1186/s12883-023-03323-2)
Supplement: Supplementary file 1 — Supplementary Material 1 [file 12883_2023_3323_MOESM1_ESM.docx]

**Additional file 1**

**Key terms for PubMed/MEDLINE search.**

| **No** | **Search Items** |
| --- | --- |
| **#1** | Alzheimer OR AD |
| **#2** | “Mild cognitive impairment” OR MCI |
| **#3** | predict* OR conver* OR  OR prognos* OR transit* OR progress* OR forecast* OR project* |
| **#4** | neuroimag* OR imag* OR MRI OR “magnetic resonance” OR  fMRI OR “functional magnetic resonance” OR sMRI OR “structural magnetic resonance” OR  PET OR “positron emission tomography” OR SPECT OR “single photon emission computed tomography” OR EEG OR electroencephalogra* OR MEG OR magnetoencephalogra* OR CT  OR “computed tomography” |
| **#5** | Search #1 AND #2 AND #3 AND #4  Limited to studies from 2017 to the date of search commencement. |
